# Altered white matter connectivity in patients with schizophrenia: An investigation using public neuroimaging data from SchizConnect

**DOI:** 10.1371/journal.pone.0205369

**Published:** 2018-10-09

**Authors:** Sung Woo Joo, Woon Yoon, Seung-Hyun Shon, Harin Kim, Saetbyeol Cha, Kee Jeong Park, Jungsun Lee

**Affiliations:** 1 Republic of Korea Navy, Donghae, Korea; 2 Department of Psychiatry, Asan Medical Center, University of Ulsan College of Medicine, Seoul, Korea; 3 Department of Psychiatry, Korean Armed Forces Capital Hospital, Bundang-gu, Korea; Chinese Academy of Sciences, CHINA

## Abstract

Several studies have produced extensive evidence on white matter abnormalities in schizophrenia (SZ). However, optimum consistency and reproducibility have not been achieved, and reported low white matter tract integrity in patients with SZ varies between studies. A whole-brain imaging study with a large sample size is needed. This study aimed to investigate white matter integrity in the corpus callosum and connections between regions of interests (ROIs) in the same hemisphere in 122 patients with SZ and 129 healthy controls with public neuroimaging data from SchizConnect. For each diffusion-weighted image (DWI), two-tensor full-brain tractography was performed; DWIs were parcellated by processing and registering T1 images with FreeSurfer and Advanced Normalization Tools. White matter query language was used to extract white matter fiber tracts. We evaluated group differences in means of diffusion measures between the patients and controls, and correlations of diffusion measures with the severity of clinical symptoms and cognitive impairment in the patients using the Positive and Negative Syndrome Scale (PANSS), a letter-number sequencing (LNS) test, vocabulary test, letter fluency test, category fluency test, and trail-making test, part A. To correct for multiple comparisons, a false discovery rate of q < 0.05 was applied. In patients with SZ, we observed significant radial diffusivity (RD) and trace (TR) increases in left thalamo-occipital tracts and the right uncinate fascicle, and a significant RD increase in the right middle longitudinal fascicle (MDLF) and the right superior longitudinal fascicle ii. Correlations were present between TR of left thalamo-occipital tracts, and the letter fluency test and the LNS test, and RD in the right MDLF and PANSS positive subscale score. However, these correlations were not significant after correction for multiple comparisons. These results indicated widespread white matter fiber tract abnormalities in patients with SZ, contributing to SZ pathophysiology.

## Introduction

Schizophrenia (SZ) is a severe and debilitating disorder characterized by disordered thought process and impaired emotional responses. The disconnection hypothesis, according to which compromised functional cerebral integration is implicated in SZ, is a leading SZ pathophysiology hypothesis [1, 2]. Structural white matter fiber tract abnormalities may underlie connectivity abnormalities, resulting in clinical symptoms and cognitive impairments [3]. Structural white matter abnormalities have been reported in patients with SZ. Consistent findings of alterations in myelin pathology, neuronal orientation and density, and oligodendrocyte aberrations in SZ have been reported [4, 5]. Post-mortem histopathological studies could be informative, but samples comprising patients of advanced age and possibly with chronic illness are a significant limitation. These studies are unable to explore white matter alterations at all stages of SZ.

Diffusion-weighted imaging (DWI) has facilitated in-vivo investigation of white matter microstructure alterations as well as the association between white matter changes and clinical symptoms [6, 7]. In 1998, DWI was used for the first time to patients with SZ [8]. Since then, a large body of evidence on white matter abnormalities in SZ has been produced. However, optimum consistency and reproducibility have not been achieved. Several factors contributing to the heterogeneity of the literature have been suggested, including methodological differences between studies, clinical presentation variability of SZ, differences in individual pathogenesis and pathophysiology, and large differences in covariates such as age, sex, chronicity, and medication [9].

To address this heterogeneity, multi-site consortia have been created to coordinate large dataset collections, including the Mind Clinical Imaging Consortium (MCIC) [10], Functional Biomedical Informatics Research Network [11–13], and North American Prodrome Longitudinal Study Consortium [14]. Due to the challenges in combining data from datasets with different protocols and data structures, these efforts have been partially successful. SchizConnect (www.schizconnect.org), a virtual database for SZ and related disorders, was built to address the issue of multiple data repositories [15, 16]. The SchizConnect system provides neuroimaging data in response to user queries, while data remain at the original sources. It mediates data sources from different studies, and provides the imaging and related data as a uniform, semantically-consistent structure by interpreting database specific terminology. Live data, being updated at each data source, can be mediated to allow access to existing datasets.

Several methods are available for diffusion tensor imaging (DTI) quantitative measurements, including voxel-based morphometry (VBM), tract-based spatial statistics (TBSS), and tractography. The VBM and TBSS methods are very popular tools for performing voxel-based DTI analyses. In these methods, all subjects’ images are co-registered to a common space, and statistical tests are performed in each co-registered voxel [17]. While these methods have a strength of allowing for automated and relatively fast analysis of entire subjects, methodological issues regarding the quality of image registration and the anatomical specificity have been reported [18, 19]. Tractography can be used to characterize entire white matter tracts and to examine white matter fascicle architecture. Using this method, white matter fiber tracts can be extracted and calculated with a measure of fiber integrity averaged over the extracted tract. Traditional fiber-tracking models cannot provide correct fiber orientations in regions where fiber bundles intersect each other because of orientation heterogeneity [20]. Fiber populations with different spatial orientations in a single voxel can cause an increase in fractional anisotropy (FA), without changes in axonal or myelin integrity [21]. The fiber-crossing problem has been a challenging issue in tractography because the proportion of white matter voxels containing crossing fibers is estimated to be ~90% [22]. Unscented Kalman filter (UKF)-based two-tensor tractography allows for the recovery of branching and crossing fibers because this tractography models the signal as a mixture of two tensors [23]. With this algorithm, Rathi et al. revealed a significant group difference in at least one diffusion measure of 740/1254 fiber bundles between patients with first-episode SZ and healthy controls (HCs) [23].

The relationship between white matter alterations and cognitive impairments in patients with SZ has been investigated with inconsistent results. Reportedly, reduced FA in the uncinate and cingulum bundles are correlated with episodic memory and executive function in chronic SZ, respectively [24]. A study in patients with recent-onset psychosis also revealed reduced FA in both uncinate fasciculi correlating with worse verbal learning/memory functioning [25]. Conversely, Samartzis et al. reported that only four of the eight studies examining associations of cognitive variables with DTI measures showed positive findings [26]. Given evidence indicating that cognitive impairments are correlated with poorer functional outcomes [27, 28], whole-brain imaging studies with larger sample size are needed to elucidate the association between white matter integrity and neurocognitive deficits.

Several studies have examined the relationship between white matter integrity alteration and clinical symptoms, reporting high variability of findings. Positive symptoms are positively related to increased FA of the inferior occipitofrontal fasciculus (IOFF), inferior longitudinal fasciculus (ILF), and superior longitudinal fasciculus (SLF) in drug-naïve first episode SZ (FES) and chronic patients [29]. However, no significant correlation was found between positive symptoms and average FA value of any above-mentioned tract in medication-naïve patients with FES [30]. Lener et al. reported that lower FA in the left ILF is associated with greater overall and positive symptoms [31]. Reported white matter tracts associated with negative symptoms vary between studies, including the corpus callosum (CC), uncinate fascicle (UF), ILF, IOFF, and anterior thalamic radiation [29, 30].

We aimed to investigate differences in diffusion indices of white matter fiber tracts between patients with SZ and HCs using UKF tractography. The analysis was focused on intra-hemispheric fiber tracts and the CC. We predicted a significant group difference in diffusion measures of white matter fiber tracts between patients with SZ and HCs. We hypothesized that clinical symptoms and cognitive impairment in patients with SZ is correlated with diffusion indices.

## Materials and methods

### Participants

We obtained three freely available samples [Center of Biomedical Research Excellence (COBRE), MCIC, and Neuromorphometry by Computer Algorithm Chicago (NMorphCH)] from SchizConnect, which comprised 140 HCs and 140 patients on the SZ spectrum encompassing SZ and schizoaffective disorder. In order to protect participant identity, all data were fully anonymized prior to our access. Ten schizoaffective patients were excluded from the 140 patients on the SZ spectrum. We visually inspected T1 and DWIs of all 270 subjects and excluded 19 subjects whose images were not eligible for further analysis. The final data set comprised 129 HC and 122 patients with SZ. Each study sample was obtained in accordance with the Declaration of Helsinki and approved by the local institutional review board committees, with participants’ written informed consent. The capacity of patients to provide informed consent was confirmed by completing a questionnaire verifying that they understood the study procedures. Parental consent was not required, as all participants were between the ages of 20 and 66. The current study was approved by the institutional review board of Asan Medical Center (IRB File No. S2017-1996-0001).

### Image acquisition

Information on scanners and acquisition parameters of structural T1 and DWIs, by sample, are summarized in S1 Table.

### Image processing

The average motion estimate of each subject was calculated using the RMSDIFF program distributed with the Functional Magnetic Resonance Imaging of the Brain (FMRIB) Software Library (FSL) (http://www.fmrib.ox.ac.uk/fsl/) [32]. The means of motion estimates in the SZ and HC groups were compared using a Welch’s test, and the SZ group had significantly higher motion estimates than that of the HC group (2.340 ± 1.986 vs. 2.010. ± 1.611, respectively; *df* = 6969.3, *t* = 7.894, *p* = < 0.001). DWIs were corrected for motion and eddy current-induced distortions, with the affine registration of all gradient volumes, with the first b = 0 volume (FLIRT: FMRIB Software, Oxford, United Kingdom) [33, 34]. After correction for the motion and eddy current-induced distortions, all images were visually inspected to eliminate images which were not eligible for further analyses. We used UKF tractography implemented in the 3D Slicer (http://www.slicer.org) [35] to perform whole-brain tractography (parameters: seeds per voxels: 10; number of tensors: two; Qm 0.001; Ql 70; Rs 0.015; record length: 1.7; step length: 0.3; and seed FA limit: 0.18) [23].

The Desikan-Killiany atlas of FreeSurfer V. 6.0 [36] was used to parcellate T1-weighted images into discrete anatomical regions. A T1-weighted image was registered into a b = 0 baseline image of DWIs with a non-linear registration method, part of the Advanced Normalization Tools [37, 38]. FreeSurfer parcellated labels were applied to derive anatomical segmentations of DWIs using the same registration transformation. Gray matter (GM)/white matter-transformed parcellated regions of interest (ROIs) on left and right sides were selected; for each structure, GM and white matter ROIs were combined into one ROI. The left (right) tract between ROIs in the same hemisphere was extracted with white matter query language [34] implemented in the 3D Slicer. The CC was also extracted based on previous studies, which reported that patients with SZ had low white matter integrity in the CC compared to controls [39, 40]. White matter query language was developed to automatically dissect white matter tracts from DWI volumes. The operation of white matter query language could be divided into two steps: (1) sets of streamlines per each anatomical region were defined, including all streamlines which had the initial or final point in the region, all streamlines traversing the region, and the streamlines traversing brain areas defined by their relative position to the region, (2) logical operations and compositions with these sets of streamlines per region were assigned to define white matter tracts. The extracted tracts (see S2 Table for information on the anatomical definitions, in white matter query language, used to extract the white matter fiber tracts) included the arcuate fascicle (AF), cingulum bundle (CB), CC, corticospinal tract (CST), external capsule (EC), ILF, internal capsule (IC), IOFF, middle longitudinal fascicle (MDLF), SLF, superior occipitofrontal fascicle, striato-frontal tract, striato-parietal tract, striato-occipital tract, thalamo-frontal tract, thalamo-parietal tract, thalamo-occipital tract, and UF. The diffusion measures [FA, axial diffusivity, radial diffusivity (RD), and trace (TR)] were calculated and averaged over each tract, according to the methods of previous reports [41–43].

### Neurocognitive tests and clinical measures

We selected neurocognitive measures that were commonly included in the three samples, which comprised the letter-number sequencing (LNS) test, vocabulary test, letter fluency test, category fluency test, and trail-making test (TMT), part A. The LNS test was used to measure verbal working memory. The LNS subtest of the Wechsler Adult Intelligence Scale–Third Edition 16 (WAIS-III) [44] was used in MCIC and NMorphCH samples, whereas the letter-number span test in the Measurement and Treatment Research to Improve Cognition in Schizophrenia consensus cognitive battery [45] was used in the COBRE sample. In COBRE and MCIC samples, general cognitive abilities and achievement were measured using the vocabulary subtest of WAIS-III, whereas the vocabulary subtest of the Wechsler Abbreviated Scale of Intelligence [46] was used in the NMorphCH sample. The letter fluency and category fluency tests assessed executive function [47, 48]. TMT, part A measured the processing speed during the attention and working memory tasks. Additionally, t-scores on the above five neurocognitive measures were obtained from the COBRE sample.

The Positive and Negative Syndrome Scale (PANSS) [49] scores in the COBRE sample and the Scale for the Assessment of Positive Symptoms (SAPS) [50] and the Scale for the Assessment of Negative Symptoms (SANS) [51] scores in the MCIC and NMorphCH samples were used to evaluate the severity of clinical symptoms. We estimated PANSS positive and negative subscale scores from SAPS and SANS scores using a validated conversion equation [52]. Clinical symptom severity was measured by these converted scores in the MCIC and NMorphCH samples and the PANSS scores in the COBRE sample.

### Statistical analysis

For comparison of continuous and categorical variables in the demographic and clinical characteristic data, a Student t-test (continuous variables with equal variance), a Welch’s test (continuous variables with unequal variance), and a chi-square test (categorical variables) were adopted. We calculated the means and standard deviations of the diffusion measures in each tract in the HC group separately for the three samples. Raw values of the patients’ diffusion measures were normalized into z-scores using the above means and standard deviations in the HC group. Outliers were defined as values below the 1.5 interquartile range (IQR) of the first quartile or above the 1.5 IQR of the third quartile of each diffusion measure in the tracts, and these were excluded from further analysis. Group differences in the z-scores of diffusion measures in tracts were compared using the Approximate Monte Carlo Fisher-Pitman Test [53, 54] with a simulation number of 100,000 because z-scores of diffusion measures were not normally distributed. To correct for multiple comparisons, a false discovery rate of q < 0.05 was applied [55]. Spearman’s rho correlations were applied to evaluate associations between the diffusion measures, and the neurocognitive measures and clinical symptom severity in patients. All statistical analyses were performed using R packages (version 3.4.3) [56, 57]. A two-tailed *p* < 0.05 was considered significant.

## Results

### Demographic and clinical characteristics

The demographic and clinical characteristics of the participants are presented in Table 1. Mean age did not differ significantly between the HC and SZ groups (35.8 ± 11.4 vs. 35.9 ± 11.0, respectively; *df* = 248.859, *t* = −0.084, *p* = 0.933). The SZ group had a larger proportion of males than the HC group (73.8% vs. 66.7%, respectively; *X*^2^ = 1.19, *p* = 0.275), but this was not statistically significant. Information on the duration of illness (15.5 ± 12.5 years) was only available from two samples (COBRE and MCIC).

**Table 1:** Demographic and clinical characteristics of participants.

Variables	Sample								Statistical tests^a^		
	COBRE		MCIC		NMorphCH		Total				
	HC	SZ	HC	SZ	HC	SZ	HC	SZ	t or χ^2^	df	p
Number of participants	74	59	19	23	36	40	129	122			
Age, mean (SD), (y)	38.2 (12.0)	38.8 (12.9)	34.8 (11.7)	34.0 (10.2)	31.3 (8.4)	32.6 (6.7)	35.8 (11.4)	35.9 (11.0)	−0.084	248.859	0.933
Male (%)	77.0	76.3	47.4	73.9	55.6	70.0	66.7	73.8	1.19		0.275
Age of onset, mean(SD), (y)		21.1 (8.3)		24.1 (6.7)		NA		22.0 (8.0)			
Duration of illness, mean(SD), (y)		17.7 (13.1)		9.9 (9.1)		NA		15.5 (12.5)			
Medication dose, mean(SD), (mg/day, olanzapine equivalent dose)^b^		17.1 (13.2)		NA		NA					
Neurocognitive tests^c^, mean(SD)											
Trail making test, part A	23.2 (7.1)	38.2 (19.3)	24.7 (7.6)	31.9 (14.2)	27.6 (11.2)	40.8 (15.7)	24.3 (8.3)	37.6 (17.4)	−7.159	149.488	<0.001
Letter fluency test	41.8 (10.7)	33.0 (11.8)	41.6 (9.9)	37.7 (10.5)	41.5 (11.1)	35.1 (12.2)	41.7 (10.6)	34.6 (11.7)	4.707	219	<0.001
Category fluency test	24.3 (4.7)	18.5 (6.5)	25.4 (3.5)	20.0 (5.1)	21.0 (5.1)	16.3 (4.3)	23.8 (4.8)	18.2 (5.7)	7.923	207.751	<0.001
Letter-number sequencing test	15.6 (3.5)	12.7 (4.4)	12.5 (1.7)	10.2 (2.1)	12.3 (2.9)	9.8 (2.9)	14.4 (3.5)	11.3 (3.8)	6.428	219	<0.001
Vocabulary test	61.2 (7.9)	51.4 (13.6)	57.2 (3.8)	45.9 (11.8)	63.0 (8.6)	54.7 (12.7)	60.9 (7.7)	51.2 (13.2)	6.620	168.213	<0.001
PANSS^d^											
Total		59.8 (16.4)		NA		NA					
Positive		15.4 (5.4)		21.1 (3.7)		27.1 (12.0)		20.3 (9.5)			
Negative		14.9 (5.5)		21.8 (3.1)		32.6 (12.1)		22.0 (11.4)			
General		29.5 (9.8)		NA		NA					

Note: COBRE (Center of Biomedical Research Excellence), MCIC (Mind Clinical Imaging Consortium), NMorphCH (Neuromorphometry by Computer Algorithm Chicago), HC (healthy controls), SZ (patients with schizophrenia), SD (standard deviation), PANSS (Positive and Negative Syndrome Scale), NA (not available).

^a^ Analyzed using a Student *t*-test (for equal variance), a Welch’s test (for unequal variance), and chi-square tests.

^b^ Antipsychotic medication dose equivalent to olanzapine at MRI scan.

^c^ Information on neurocognitive measures was available from 221 of 251 participants.

^d^ PANSS total and three subscale scores were only obtained from COBRE sample. SAPS and SANS scores in the MCIC and NMorphCH samples were converted to PANSS positive and negative subscale scores.

Information on neurocognitive measures was available for 221 of the 251 participants. There were statistically significant differences in all neurocognitive measures between the two groups. The SZ group exhibited significantly lower scores on the letter fluency test, category fluency test, LNS test, and vocabulary test (*df* = 219, *t* = 4.707, *p* < 0.001; *df* = 207.751, *t* = 7.923, *p* < 0.001; *df* = 219, *t* = 6.428, *p* < 0.001; and *df* = 168.213, *t* = 6.620, *p* < 0.001, respectively). The SZ group mean score on TMT, part A was significantly higher than the HC group mean score (*df* = 149.488, *t* = −7.159, *p* < 0.001). Table 1 presents means of the PANSS total scores and three subscale scores in the COBRE sample, converted PANSS positive and negative subscale scores in the MCIC and NMorphCH samples, and olanzapine equivalent dose [58] of the SZ group in the COBRE sample.

### Comparisons of diffusion MRI measures

There were no significant differences in the diffusion measures of the CC between the two groups (AD: *z* = −0.946, *p* = 0.611, FDR corrected; FA: *z* = −0.103, *p* = 0.939, FDR corrected; RD: *z* = −0.980, *p* = 0.598, FDR corrected; TR: *z* = −0.868, *p* = 0.653, FDR corrected). Group differences in the diffusion measures of the tracts in the left and right hemispheres between the two groups are shown in Table 2 (left hemisphere) and Table 3 (right hemisphere). The SZ group had significantly higher RD and TR of left thalamo-occipital tracts (RD: *z* = −3.642, *p* = 0.016, FDR corrected; TR: *z* = −3.125, *p* = 0.043, FDR corrected) and UF (RD: *z* = −4.081, *p* = 0.002, FDR corrected; TR: *z* = −3.393, *p* = 0.035, FDR corrected) than the HC group. In the SZ group, RD of the right MDLF (*z* = −3.093, *p* = 0.046, FDR corrected) and the right SLF ii (*z* = −3.142, *p* = 0.043, FDR corrected) were significantly higher than the HC group.

**Table 2:** Group differences in the diffusion measures of tracts in the left hemisphere^a^.

Structure	Diffusion measure							
	AD		FA		RD		TR	
	*z*	*p*^b^	*z*	*p*^b^	*z*	*p*^b^	*z*	*p*^b^
left AF	0.313	0.755	0.395	0.693	−1.575	0.116	−0.285	0.776
left CB	0.104	0.918	1.901	0.056	−1.634	0.102	−1.064	0.291
left CST	0.192	0.847	0.515	0.610	−0.403	0.691	0.663	0.508
left EC	0.756	0.451	2.04	0.041	−2.728	0.006	−1.410	0.159
left ILF	0.791	0.427	2.552	0.011	−2.910	0.004	−1.800	0.070
left IC	0.180	0.856	1.749	0.078	-2.030	0.043	-1.155	0.247
left IOFF	0.309	0.762	0.512	0.611	−0.753	0.455	−0.421	0.684
left MDLF	1.261	0.207	2.594	0.009	−2.869	0.004	−1.086	0.281
left SLF i	1.578	0.115	1.681	0.092	−1.863	0.062	−1.525	0.129
left SLF ii	−0.263	0.824	2.243	0.023	−2.531	0.008	−1.929	0.053
left SLF iii	1.960	0.049	−0.331	0.745	0.705	0.482	0.422	0.673
left SOFF	0.199	0.843	−0.239	0.811	1.528	0.127	−0.199	0.863
left striato-frontal	0.841	0.401	1.061	0.289	−1.796	0.073	−0.256	0.797
left striato-occipital	−0.973	0.334	0.270	0.788	−1.588	0.113	−1.430	0.153
left striato-parietal	1.714	0.088	1.183	0.238	−0.459	0.649	0.677	0.500
left thalamo-frontal	0.854	0.396	−0.641	0.560	−0.499	0.619	0.104	0.920
left thalamo-occipital	−1.676	0.094	2.505	0.012	−3.642	<0.001*	−3.125	0.001*
left thalamo-parietal	0.145	0.885	0.491	0.624	−0.420	0.677	−0.257	0.797
left UF	−1.896	0.058	−0.003	0.998	−1.461	0.143	−1.535	0.125

Note: AF (arcuate fascicle), CB (cingulum bundle), CST (corticospinal tract), EC (external capsule), ILF (inferior longitudinal fascicle), IC (internal capsule), IOFF (inferior occipitofrontal fascicle), MDLF (middle longitudinal fascicle), SLF (superior longitudinal fascicle), SOFF (superior occipitofrontal fascicle), UF (uncinate fascicle), AD (axial diffusivity), FA (fractional anisotropy), RD (radial diffusivity), TR (trace).

^a^ Approximate Monte Carlo Fisher–Pitman tests were performed to reveal group differences in the z-scores of the diffusion measures of tracts in the left hemisphere.

^b^ Uncorrected p-values.

* False discovery rate-adjusted p-value less than 0.05.

**Table 3:** Group differences in the diffusion measures of tracts in the right hemisphere^a^.

Structure	Diffusion measure							
	AD		FA		RD		TR	
	*z*	*p*^b^	*z*	*p*^b^	*z*	*p*^b^	*z*	*p*^b^
right AF	-0.192	0.846	0.162	0.872	-1.246	0.213	-0.898	0.373
right CB	1.819	0.068	0.466	0.680	-1.409	0.158	0.430	0.670
right CST	-0.062	0.950	0.861	0.390	-1.276	0.201	-0.334	0.739
right EC	-0.140	0.890	0.521	0.604	-1.785	0.074	-1.120	0.264
right ILF	0.528	0.598	2.177	0.029	-2.259	0.024	-1.697	0.090
right IC	0.782	0.436	1.407	0.159	-0.952	0.343	-0.280	0.781
right IOFF	-0.912	0.364	0.458	0.656	-2.568	0.010	-1.619	0.105
right MDLF	1.079	0.280	2.66	0.007	-3.093	0.002*	-2.210	0.026
right SLF i	0.526	0.603	1.255	0.211	-1.540	0.124	-1.405	0.164
right SLF ii	1.292	0.197	1.894	0.056	-3.142	0.001*	-1.583	0.114
right SLF iii	1.226	0.220	2.585	0.010	-1.814	0.070	-0.862	0.392
right SOFF	0.435	0.666	1.124	0.269	-1.295	0.198	-1.246	0.213
right striato-frontal	1.756	0.078	1.421	0.156	-1.106	0.270	0.292	0.770
right striato-occipital	0.332	0.741	-0.341	0.732	0.352	0.724	0.464	0.647
right striato-parietal	1.242	0.215	2.341	0.019	-1.608	0.109	-1.168	0.245
right thalamo-frontal	0.836	0.404	0.745	0.525	-2.218	0.025	-1.439	0.081
right thalamo-occipital	0.577	0.574	1.882	0.059	-2.006	0.043	-1.007	0.328
right thalamo-parietal	-0.694	0.485	0.694	0.488	-1.293	0.196	-0.827	0.407
right UF	0.029	0.977	2.633	0.008	-4.081	<0.001*	-3.393	0.001*

Note: AF (arcuate fascicle), CB (cingulum bundle), CST (corticospinal tract), EC (external capsule), ILF (inferior longitudinal fascicle), IC (internal capsule), IOFF (inferior occipitofrontal fascicle), MDLF (middle longitudinal fascicle), SLF (superior longitudinal fascicle), SOFF (superior occipitofrontal fascicle), UF (uncinate fascicle), AD (axial diffusivity), FA (fractional anisotropy), RD (radial diffusivity), TR (trace).

^a^ Approximate Monte Carlo Fisher–Pitman tests were performed to reveal group differences in the z-scores of the diffusion measures of tracts in the right hemisphere.

^b^ Uncorrected p-values.

* False discovery rate-adjusted p-value less than 0.05.

We performed the same analyses with age and sex as covariates and identified the same group differences in diffusion measures of white matter fiber tracts (left thalamo-occipital tracts: RD; *z* = −3.631, *p* = 0.021, FDR corrected; TR; *z* = −3.121, *p* = 0.040, FDR corrected; right MDLF: RD; *z* = −2.971, *p* = 0.049, FDR corrected; right SLF ii: RD; *z* = −3.132, *p* = 0.040, FDR corrected; right UF: RD; *z* = −4.019, *p* = 0.007, FDR corrected; and TR; *z* = −3.383, *p* = 0.029, FDR corrected).

### Clinical correlates of diffusion MRI measures

We selected diffusion measures of tracts that showed significant group differences, after correction for multiple comparisons. In the SZ group, correlations between the raw values of the above diffusion measures and the neurocognitive measures were evaluated (Table 4). There were Spearman’s rho correlations between TR in the left thalamo-occipital tracts, and the letter fluency test (*r* = −0.236, uncorrected *p* = 0.018) and the LNS test (*r* = −0.198, uncorrected *p* = 0.048). However, these two correlations did not survive after correction for multiple comparisons. Using the COBRE sample, we evaluated t-score associations of the neurocognitive measures with the diffusion measures. We observed correlation between RD in the right UF and TMT, part A scores (*r* = −0.326, uncorrected *p* = 0.019), which did not remain significant after correction for multiple comparisons. There was an association between RD in the right MDLF and PANSS positive subscale score (*r* = 0.212, uncorrected *p* = 0.023), which did not reach statistical significance after correction for multiple comparisons. With information about medication dose at the MRI scan in COBRE sample, we examined associations of olanzapine equivalent dose with diffusion measures. Associations of olanzapine equivalent dose, and RD in the right MDLF and SLF ii (*r* = 0.285, uncorrected *p* = 0.030; *r* = 0.294, uncorrected *p* = 0.026, respectively) did not remain significant after correction for multiple comparisons.

**Table 4:** Correlations between the diffusion measures and neurocognitive characteristics of the participants^a^.

Structure	Diffusion measure	Trail making test, part A		Letter fluency test		Category fluency test		Letter number sequencing test		Vocabulary test	
		*r*	p*	*r*	p*	*r*	p*	*r*	p*	*r*	p*
left thalamo-occipital	RD	−0.013	0.894	−0.162	0.107	−0.027	0.788	−0.194	0.053	−0.135	0.18
left thalamo-occipital	TR	0.037	0.711	−0.236	0.018	−0.066	0.516	−0.198	0.048	−0.137	0.176
right MDLF	RD	−0.103	0.307	−0.149	0.138	−0.144	0.152	−0.146	0.146	−0.174	0.082
right SLF ii	RD	−0.076	0.447	−0.154	0.123	−0.023	0.818	−0.101	0.311	−0.103	0.301
right UF	RD	0.01	0.921	−0.156	0.12	−0.077	0.444	−0.005	0.964	−0.171	0.088
right UF	TR	−0.056	0.578	−0.128	0.201	−0.019	0.848	−0.025	0.805	−0.17	0.09

Note: MDLF (middle longitudinal fascicle), SLF (superior longitudinal fascicle), UF (uncinate fascicle), RD (radial diffusivity), TR (trace).

^a^ Spearman’s rho correlations were conducted to evaluate associations between the raw values of the diffusion measures and neurocognitive characteristics.

* Uncorrected p-values.

## Discussion

We examined the integrity of CC and white matter fiber tracts connecting ROIs within the same hemisphere in patients with SZ. We used UKF tractography to extract white matter fiber tracts with public neuroimaging data from SchizConnect. To the best of our knowledge, this is the first study to investigate white matter fiber tracts in patients with SZ using public neuroimaging data from multiple data repositories. We found significant RD and TR increases in left thalamo-occipital tracts and the right UF, and a significant RD increase in the right MDLF and right SLF ii in patients with SZ. There were correlations between TR of left thalamo-occipital tracts, and the letter fluency test and the LNS test, and RD in the right MDLF and PANSS positive subscale score. However, these correlations were not statistically significant after correction for multiple comparisons.

FA is an index derived from DTI for evaluating anisotropy in white matter. FA abnormalities in patients with demyelinating disease were reported in early DTI studies [59]. Therefore, FA was considered a proxy for myelin integrity. Although myelination contributes to FA, other factors, including the axon itself and tract geometry, can influence FA [21, 60]. Relatively pure deficits in myelin can cause a modest increase in RD, with anisotropy preservation [21]. RD may be a more useful diffusion index than FA to reflect subtle impairment of myelin integrity and for examining white matter fiber integrity in early-course SZ. TR, as an index of the magnitude of water diffusion, has been used to quantify white matter abnormalities in previous DTI studies [42]. While RD change is reported to be associated with myelin neuropathology [61], TR increase has been attributed to general microstructural white matter pathologies. Our results showed RD and/or TR increases in patients with SZ, with no significant FA decreases. A previous study suggested RD as a demyelination surrogate [62]. However, a study with simulated data demonstrated that a simplistic biological RD interpretation should be avoided because of inter-individual variation in underlying tissue and interactions between anatomical and scanning parameters [63]. Neurobiological correlates of RD abnormality with SZ need to be studied further.

We showed that RD and TR of the left thalamo-occipital tract in the SZ group were significantly increased compared with the HC group. Contrary to our findings, several studies on the connectivity of thalamo-cortical networks in patients with SZ have reported that patients with SZ show no significant differences in functional or structural connectivity of the occipital cortex with the thalamus compared with HCs [64–66]. Conversely, patients with SZ display significantly decreased FA in optic radiations compared with HCs [67, 68]. Optic radiation structure and correlations of structure measures with visual masking performance in patients with SZ were investigated [69]. Although there are no significant differences in diffusion indices or tract volume between the patients and HCs, a correlation between the tract volume of optic radiations and visual masking performance is found in patients with SZ. Visual perception dysfunction in patients with SZ has been well-characterized in several studies [70–72]. Our findings suggest that impairment of the integrity of optic radiations and structural thalamo-occipital connectivity contributes visual perception dysfunction neural bases in patients with SZ.

MDLF was initially defined as a long association fiber tract connecting the superior temporal gyrus and temporal pole with the angular gyrus [73]. Patients with chronic SZ have a significant mean FA decrease in MDLF of both hemispheres compared with HCs [74]. Consistent with these results, we showed that the SZ group had significantly higher RD of the right MDLF than the HC group. Although the function of MDLF remains unclear, MDLF may be associated with attention, language [75], and functions connected with high-level auditory [76] and visual processing [77]. Six major fiber connections of MDLF, including temporo-parietal and temporo-occipital connections, were reported, expanding the human MDLF classification [77]. Given the extensive connectivity of the human MDLF to various cortical structures, it is possible that MDLF is implicated in SZ pathophysiology.

Impaired white matter integrity within the prefrontal, temporal, and parietal lobes in patients with SZ has been reported, along with abnormalities within fiber bundles such as UF and SLF that are connecting these regions [6, 9]. UF and SLF abnormalities have been reported in patients with FES, chronic SZ, and drug-naïve SZ [9, 26, 78, 79]. Our study adds to this literature by showing impaired white matter integrity of the right UF and SLF in patients with SZ.

Verbal fluency is consistently impaired over time and is related to these patients’ clinical and functional outcomes [80]. Verbal fluency tests are used to measure executive function; the performance of a patient with SZ on these tests can be predicted by their working memory or processing speed, depending on cognitive impairment severity [81]. Working memory deficit, as a core neurocognitive impairment, has been extensively reported and studied in patients with SZ [82–84]. The LNS test is used to measure executive function working memory, and it requires transient working memory as well as additional mental manipulation of the information [85]. Here the association between verbal fluency, the LNS test, and the diffusion measures did not reach statistical significance. It may be possible that various functions required for verbal fluency and the LNS test are related to the integrity of several white matter fiber tracts, rather than a specific white matter fiber tract. Further studies concerning the entire brain connectivity may be needed to address associations of structural measures with neurocognitive variables.

The relationship of white matter alterations and clinical symptoms in SZ has been examined with inconsistent evidence. Viher et al. reported that negative symptoms are related to white matter microstructure of the prefrontal and right temporal lobes, but no association of white matter microstructure and positive symptoms was found [86]. Conversely, Lener et al. showed that lower FA in the genu is associated with greater positive symptom and none of the correlations with negative symptoms are significant [31]. We found the correlation of RD in the right MDLF and PANSS positive subscale score; however, the correlation did not survive after correction for multiple comparisons. The reason for this negative finding may be heterogeneity in illness duration of the SZ group, medication effects on symptom severity, and different scales for measuring symptom severity in the three samples.

The present study has several methodological limitations. First, we did not separate the patients into FES and chronic SZ groups. There are different patterns of white matter impairment among individuals at high clinical risk of psychosis, those with FES, and those with chronic SZ [9]. Particularly, there is disruption in the integrity of the white matter fiber tracts in the right hemisphere of patients with FES, but not chronic SZ [87, 88]. Although this separation may have revealed different patterns of disruption in white matter fiber tracts between patients with FES and chronic SZ, a smaller sample size derived by dividing the patients into FES and chronic groups may have reduced the statistical power of this study. Second, antipsychotics use was not considered because information about medication use was not available in all three samples. Findings on the effects of antipsychotics on diffusion measures in patients with SZ remain inconclusive. Specifically, while a few studies have shown no association between antipsychotics use and changes in diffusion measures [89, 90], other studies have revealed such effects [91–93]. Further studies are needed to investigate this association. Third, changes within the white matter of patients with SZ may be confounded by age and sex. Accelerated FA decline with age in patients with SZ has been reported [94]; this effect is more prominent in specific tracts that mature later in life [95]. A relationship between neurodevelopmental changes in white matter and age has been suggested [96]. Although, in the present study, all the group differences in diffusion measures remained significant after adjustment for age and sex, the effect of age and sex on diffusion measures could not be entirely disregarded. Fourth, three different samples were included. Although the raw values of the patients’ diffusion measures were normalized, it was not possible to entirely disregard the effect of different protocol parameters on the calculated diffusion measures. A certain sample may have influenced the positive findings of this study. We conducted the same analyses separately for the three samples, and there were no significant group differences in any diffusion measure of tracts after correction for multiple comparisons (see S3 Table for details on the group differences in the diffusion measures, by sample). We also performed analyses to compare the means of the diffusion measures among the three samples in the SZ and HC groups. The diffusion measures of some fiber tracts had significant sample-differences among three samples in the SZ group; however, the diffusion measures which showed significant group-differences between the SZ and HC groups did not have significant sample-differences in the SZ group. A larger sample size, achieved by combining the three samples, provided greater statistical power that contributed to this study’s positive findings. Fifth, the angular resolution of DWI acquired in the MCIC sample was relatively low. DWI data having low angular resolution may lead to inaccurate fitting during extraction of white matter fiber tracts with a two-tensor model [97]. To account for this problem, the MCIC sample was excluded, and the same analyses were performed again. The results showed that the SZ group had significantly higher RD of left thalamo-occipital tracts (RD: *z* = −3.280, *p* = 0.034, FDR corrected) and RD and TR of right UF (RD: *z* = −3.740, *p* = 0.018, FDR corrected; TR: *z* = −3.513, *p* = 0.029, FDR corrected) than the HC group. Three of the six significant diffusion measures remained significant. Future research that considers the effect of low angular resolution in the two-tensor model is needed to verify the significance of findings in this study. Sixth, we did not account for the effect of motion artifacts on the diffusion measures. A previous study [98] reported that artifacts from even small head motion can induce spurious group differences in the diffusion measures. We conducted the same analyses with covariates of age, sex, and motion estimate. The results showed no significant group differences in the diffusion measures after correcting for multiple comparisons (left thalamo-occipital tracts: RD; *z* = 1.932, uncorrected *p* = 0.052, TR; *z* = 1.934, uncorrected *p* = 0.054, right MDLF: RD; *z* = 1.783, uncorrected *p* = 0.074, right SLF ii: RD; *z* = −2.115, uncorrected *p* = 0.034, right UF: RD; *z* = −2.183, uncorrected *p* = 0.029, TR; *z* = 2.099, uncorrected *p* = 0.036). This linear regression approach is somewhat arbitrary because the diffusion measures are not linear with respect to motion parameters [98]. The motion estimates used in our study were skewed, therefore a statistic, instead of mean and standard deviation, was required to reflect central tendency and dispersion of the motion estimates. Other motion measures which can capture within-volume motion were also needed to comprehensively reflect motion artifacts, however, we were not able to obtain such motion measures in this study. Future research that comprehensively evaluates the influence of motion artifacts on the diffusion measures is needed to validate our significant findings.

Disruption of the white matter integrity in patients with SZ is seen in the left thalamo-occipital tracts, right MDLF, right SLF ii, and right UF. These results indicate widespread white matter fiber tract abnormalities in patients with SZ, which is consistent with the disconnection hypothesis of SZ. Further studies are needed to elucidate the association between diffusion measures and clinical symptoms of SZ.

## Supporting information

S1 Table Scanners and imaging parameters by sample. COBRE (Center of Biomedical Research Excellence), MCIC (Mind Clinical Imaging Consortium), NmorphCH (Neuromorphometry by Computer Algorithm Chicago), DWI (diffusion weighted imaging).
(DOCX)

S2 Table Anatomical definitions of white matter query language. AF (arcuate fascicle), CB (cingulum bundle), CC (corpus callosum), CST (corticospinal tract), EC (external capsule), ILF (inferior longitudinal fascicle), IC (internal capsule), IOFF (inferior occipitofrontal fascicle), MDLF (middle longitudinal fascicle), SLF (superior longitudinal fascicle), SOFF (superior occipitofrontal fascicle), UF (uncinate fascicle).
(DOCX)

S3 Table Group differences in the diffusion measures of tracts in the Center of Biomedical Research Excellence (S3.1), Mind Clinical Imaging Consortium (S3.2), Neuromorphometry by Computer Algorithm Chicago (S3.3) samples. AF (arcuate fascicle), CB (cingulum bundle), CST (corticospinal tract), EC (external capsule), ILF (inferior longitudinal fascicle), IC (internal capsule), IOFF (inferior occipitofrontal fascicle), MDLF (middle longitudinal fascicle), SLF (superior longitudinal fascicle), SOFF (superior occipitofrontal fascicle), UF (uncinate fascicle), AD (axial diffusivity), FA (fractional anisotropy), RD (radial diffusivity), TR (trace).
(DOCX)

## References

[1] Friston KJ. The disconnection hypothesis. Schizophrenia research. 1998;30(2):115–25. Epub 1998/04/29. . 9549774 10.1016/s0920-9964(97)00140-0

[2] Schmitt A, Hasan A, Gruber O, Falkai P. Schizophrenia as a disorder of disconnectivity. European archives of psychiatry and clinical neuroscience. 2011;261 Suppl 2:S150–4. Epub 2011/08/26. 10.1007/s00406-011-0242-2 . 21866371 PMC3207137

[3] Walterfang M, Velakoulis D, Whitford TJ, Pantelis C. Understanding aberrant white matter development in schizophrenia: an avenue for therapy? Expert Rev Neurother. 2011;11(7):971–87. Epub 2011/07/05. 10.1586/ern.11.76 . 21721915

[4] Segal D, Koschnick JR, Slegers LH, Hof PR. Oligodendrocyte pathophysiology: a new view of schizophrenia. The international journal of neuropsychopharmacology / official scientific journal of the Collegium Internationale Neuropsychopharmacologicum. 2007;10(4):503–11. Epub 2007/02/13. 10.1017/s146114570600722x . 17291369

[5] Konrad A, Winterer G. Disturbed structural connectivity in schizophrenia primary factor in pathology or epiphenomenon? Schizophrenia bulletin. 2008;34(1):72–92. Epub 2007/05/09. 10.1093/schbul/sbm034 . 17485733 PMC2632386

[6] Kubicki M, McCarley R, Westin CF, Park HJ, Maier S, Kikinis R, et al A review of diffusion tensor imaging studies in schizophrenia. Journal of psychiatric research. 2007;41(1–2):15–30. 10.1016/j.jpsychires.2005.05.005 . 16023676 PMC2768134

[7] Shenton ME, Whitford TJ, Kubicki M. Structural neuroimaging in schizophrenia: from methods to insights to treatments. Dialogues Clin Neurosci. 2010;12(3):317–32. . 20954428 10.31887/DCNS.2010.12.3/mshenton PMC3181976

[8] Buchsbaum MS, Tang CY, Peled S, Gudbjartsson H, Lu D, Hazlett EA, et al MRI white matter diffusion anisotropy and PET metabolic rate in schizophrenia. Neuroreport. 1998;9(3):425–30. . 9512384 10.1097/00001756-199802160-00013

[9] Wheeler AL, Voineskos AN. A review of structural neuroimaging in schizophrenia: from connectivity to connectomics. Front Hum Neurosci. 2014;8:653 10.3389/fnhum.2014.00653 . 25202257 PMC4142355

[10] Gollub RL, Shoemaker JM, King MD, White T, Ehrlich S, Sponheim SR, et al The MCIC collection: a shared repository of multi-modal, multi-site brain image data from a clinical investigation of schizophrenia. Neuroinformatics. 2013;11(3):367–88. 10.1007/s12021-013-9184-3 . 23760817 PMC3727653

[11] Helmer KG, Ambite JL, Ames J, Ananthakrishnan R, Burns G, Chervenak AL, et al Enabling collaborative research using the Biomedical Informatics Research Network (BIRN). J Am Med Inform Assoc. 2011;18(4):416–22. 10.1136/amiajnl-2010-000032 . 21515543 PMC3128398

[12] Friedman L, Stern H, Brown GG, Mathalon DH, Turner J, Glover GH, et al Test-retest and between-site reliability in a multicenter fMRI study. Hum Brain Mapp. 2008;29(8):958–72. 10.1002/hbm.20440 . 17636563 PMC3670112

[13] Keator DB, Grethe JS, Marcus D, Ozyurt B, Gadde S, Murphy S, et al A national human neuroimaging collaboratory enabled by the Biomedical Informatics Research Network (BIRN). IEEE Trans Inf Technol Biomed. 2008;12(2):162–72. . 18348946 10.1109/TITB.2008.917893 PMC2763494

[14] Addington J, Cadenhead KS, Cornblatt BA, Mathalon DH, McGlashan TH, Perkins DO, et al North American Prodrome Longitudinal Study (NAPLS 2): overview and recruitment. Schizophrenia research. 2012;142(1–3):77–82. 10.1016/j.schres.2012.09.012 . 23043872 PMC3502644

[15] Ambite JL, Tallis M, Alpert K, Keator DB, King M, Landis D, et al SchizConnect: Virtual Data Integration in Neuroimaging. Data Integr Life Sci. 2015;9162:37–51. 10.1007/978-3-319-21843-4_4 . 26688837 PMC4682903

[16] Wang L, Alpert KI, Calhoun VD, Cobia DJ, Keator DB, King MD, et al SchizConnect: Mediating neuroimaging databases on schizophrenia and related disorders for large-scale integration. Neuroimage. 2016;124(Pt B):1155–67. 10.1016/j.neuroimage.2015.06.065 . 26142271 PMC4651768

[17] Smith SM, Jenkinson M, Johansen-Berg H, Rueckert D, Nichols TE, Mackay CE, et al Tract-based spatial statistics: voxelwise analysis of multi-subject diffusion data. Neuroimage. 2006;31(4):1487–505. 10.1016/j.neuroimage.2006.02.024 . 16624579

[18] Melonakos ED, Shenton ME, Rathi Y, Terry DP, Bouix S, Kubicki M. Voxel-based morphometry (VBM) studies in schizophrenia-can white matter changes be reliably detected with VBM? Psychiatry research. 2011;193(2):65–70. 10.1016/j.pscychresns.2011.01.009 . 21684124 PMC3382976

[19] Bach M, Laun FB, Leemans A, Tax CM, Biessels GJ, Stieltjes B, et al Methodological considerations on tract-based spatial statistics (TBSS). Neuroimage. 2014;100:358–69. 10.1016/j.neuroimage.2014.06.021 . 24945661

[20] Alexander AL, Hasan KM, Lazar M, Tsuruda JS, Parker DL. Analysis of partial volume effects in diffusion-tensor MRI. Magn Reson Med. 2001;45(5):770–80. Epub 2001/04/27. . 11323803 10.1002/mrm.1105

[21] Alba-Ferrara LM, de Erausquin GA. What does anisotropy measure? Insights from increased and decreased anisotropy in selective fiber tracts in schizophrenia. Front Integr Neurosci. 2013;7:9 Epub 2013/03/14. 10.3389/fnint.2013.00009 . 23483798 PMC3593197

[22] Jeurissen B, Leemans A, Tournier JD, Jones DK, Sijbers J. Investigating the prevalence of complex fiber configurations in white matter tissue with diffusion magnetic resonance imaging. Hum Brain Mapp. 2013;34(11):2747–66. Epub 2012/05/23. 10.1002/hbm.22099 . 22611035 PMC6870534

[23] Rathi Y, Kubicki M, Bouix S, Westin CF, Goldstein J, Seidman L, et al Statistical analysis of fiber bundles using multi-tensor tractography: application to first-episode schizophrenia. Magn Reson Imaging. 2011;29(4):507–15. 10.1016/j.mri.2010.10.005 . 21277725 PMC3078978

[24] Nestor PG, Kubicki M, Niznikiewicz M, Gurrera RJ, McCarley RW, Shenton ME. Neuropsychological disturbance in schizophrenia: a diffusion tensor imaging study. Neuropsychology. 2008;22(2):246–54. 10.1037/0894-4105.22.2.246 . 18331167 PMC2791789

[25] Szeszko PR, Robinson DG, Ashtari M, Vogel J, Betensky J, Sevy S, et al Clinical and neuropsychological correlates of white matter abnormalities in recent onset schizophrenia. Neuropsychopharmacology: official publication of the American College of Neuropsychopharmacology. 2008;33(5):976–84. 10.1038/sj.npp.1301480 . 17581532

[26] Samartzis L, Dima D, Fusar-Poli P, Kyriakopoulos M. White matter alterations in early stages of schizophrenia: a systematic review of diffusion tensor imaging studies. Journal of neuroimaging: official journal of the American Society of Neuroimaging. 2014;24(2):101–10. Epub 2013/01/16. 10.1111/j.1552-6569.2012.00779.x . 23317110

[27] Bowie CR, Harvey PD. Cognitive deficits and functional outcome in schizophrenia. Neuropsychiatric disease and treatment. 2006;2(4):531–6. . 19412501 10.2147/nedt.2006.2.4.531 PMC2671937

[28] Green MF. Cognitive impairment and functional outcome in schizophrenia and bipolar disorder. The Journal of clinical psychiatry. 2006;67 Suppl 9:3–8; discussion 36–42. . 16965182

[29] Canu E, Agosta F, Filippi M. A selective review of structural connectivity abnormalities of schizophrenic patients at different stages of the disease. Schizophrenia research. 2015;161(1):19–28. Epub 2014/06/05. 10.1016/j.schres.2014.05.020 . 24893909

[30] Sun H, Lui S, Yao L, Deng W, Xiao Y, Zhang W, et al Two Patterns of White Matter Abnormalities in Medication-Naive Patients With First-Episode Schizophrenia Revealed by Diffusion Tensor Imaging and Cluster Analysis. JAMA Psychiatry. 2015;72(7):678–86. Epub 2015/05/21. 10.1001/jamapsychiatry.2015.0505 . 25993492

[31] Lener MS, Wong E, Tang CY, Byne W, Goldstein KE, Blair NJ, et al White matter abnormalities in schizophrenia and schizotypal personality disorder. Schizophrenia bulletin. 2015;41(1):300–10. Epub 2014/06/26. 10.1093/schbul/sbu093 . 24962608 PMC4266294

[32] Jenkinson M, Beckmann CF, Behrens TE, Woolrich MW, Smith SM. Fsl. Neuroimage. 2012;62(2):782–90. 10.1016/j.neuroimage.2011.09.015 . 21979382

[33] Jenkinson M, Bannister P, Brady M, Smith S. Improved optimization for the robust and accurate linear registration and motion correction of brain images. Neuroimage. 2002;17(2):825–41. . 12377157 10.1016/s1053-8119(02)91132-8

[34] Jenkinson M, Smith S. A global optimisation method for robust affine registration of brain images. Med Image Anal. 2001;5(2):143–56. . 11516708 10.1016/s1361-8415(01)00036-6

[35] Fedorov A, Beichel R, Kalpathy-Cramer J, Finet J, Fillion-Robin JC, Pujol S, et al 3D Slicer as an image computing platform for the Quantitative Imaging Network. Magn Reson Imaging. 2012;30(9):1323–41. 10.1016/j.mri.2012.05.001 . 22770690 PMC3466397

[36] Fischl B, Salat DH, Busa E, Albert M, Dieterich M, Haselgrove C, et al Whole brain segmentation: automated labeling of neuroanatomical structures in the human brain. Neuron. 2002;33(3):341–55. . 11832223 10.1016/s0896-6273(02)00569-x

[37] Avants BB, Tustison NJ, Song G, Cook PA, Klein A, Gee JC. A reproducible evaluation of ANTs similarity metric performance in brain image registration. Neuroimage. 2011;54(3):2033–44. 10.1016/j.neuroimage.2010.09.025 . 20851191 PMC3065962

[38] Avants BB, Yushkevich P, Pluta J, Minkoff D, Korczykowski M, Detre J, et al The optimal template effect in hippocampus studies of diseased populations. Neuroimage. 2010;49(3):2457–66. 10.1016/j.neuroimage.2009.09.062 . 19818860 PMC2818274

[39] Fitzsimmons J, Kubicki M, Shenton ME. Review of functional and anatomical brain connectivity findings in schizophrenia. Current opinion in psychiatry. 2013;26(2):172–87. 10.1097/YCO.0b013e32835d9e6a . 23324948

[40] Balevich EC, Haznedar MM, Wang E, Newmark RE, Bloom R, Schneiderman JS, et al Corpus callosum size and diffusion tensor anisotropy in adolescents and adults with schizophrenia. Psychiatry research. 2015;231(3):244–51. 10.1016/j.pscychresns.2014.12.005 . 25637358 PMC4363270

[41] Papadakis NG, Xing D, Houston GC, Smith JM, Smith MI, James MF, et al A study of rotationally invariant and symmetric indices of diffusion anisotropy. Magn Reson Imaging. 1999;17(6):881–92. . 10402595 10.1016/s0730-725x(99)00029-6

[42] Alexander AL, Lee JE, Lazar M, Field AS. Diffusion tensor imaging of the brain. Neurotherapeutics. 2007;4(3):316–29. 10.1016/j.nurt.2007.05.011 . 17599699 PMC2041910

[43] Sommer IE, de Kort GA, Meijering AL, Dazzan P, Hulshoff Pol HE, Kahn RS, et al How frequent are radiological abnormalities in patients with psychosis? A review of 1379 MRI scans. Schizophrenia bulletin. 2013;39(4):815–9. Epub 2012/03/15. 10.1093/schbul/sbs037 . 22416264 PMC3686442

[44] Wechsler D. Wechsler adult intelligence scale. 3rd ed San Antonio, TX: Psychological Corporation; 1997.

[45] Nuechterlein KH, Green MF, Kern RS, Baade LE, Barch DM, Cohen JD, et al The MATRICS Consensus Cognitive Battery, part 1: test selection, reliability, and validity. The American journal of psychiatry. 2008;165(2):203–13. 10.1176/appi.ajp.2007.07010042 . 18172019

[46] Wechsler D. Wechsler Abbreviated Scale of Intelligence (WASI) manual: Psychological Corporation; San Antonio, TX; 1999.

[47] Benton AL. Multilingual Aphasia Examination. Iowa: University of Iowa 1976.

[48] Benton AL, Hamsher Kd, Sivan AB. Multilingual aphasia examination (MAE). 3rd ed Iowa: Psychological Assessment Resources; 1984.

[49] Kay SR, Fiszbein A, Opler LA. The positive and negative syndrome scale (PANSS) for schizophrenia. Schizophrenia bulletin. 1987;13(2):261–76. . 3616518 10.1093/schbul/13.2.261

[50] Andreasen N. The Scale for the Assessment of Positive Symptoms (SAPS). Iowa City, Iowa: Unversity of Iowa; 1984.

[51] Andreasen N. The Scale for the Assessment of Negative Symptoms (SANS). Iowa City, Iowa: Unversity of Iowa; 1984.

[52] van Erp TG, Preda A, Nguyen D, Faziola L, Turner J, Bustillo J, et al Converting positive and negative symptom scores between PANSS and SAPS/SANS. Schizophrenia research. 2014;152(1):289–94. Epub 2013/12/18. 10.1016/j.schres.2013.11.013 . 24332632 PMC3966195

[53] Kaiser J. An exact and a Monte Carlo proposal to the Fisher–Pitman permutation tests for paired replicates and for independent samples. Stata Journal. 2007;7(3):402–12.

[54] Berry KJ, Mielke PW Jr, Mielke HW. The Fisher-Pitman permutation test: an attractive alternative to the F test. Psychological reports. 2002;90(2):495–502. 10.2466/pr0.2002.90.2.495 12061589

[55] Benjamini Y, Hochberg Y. Controlling the false discovery rate: a practical and powerful approach to multiple testing. Journal of the royal statistical society Series B (Methodological). 1995;57(1):289–300.

[56] R Core Team. R: A language and environment for statistical computing. Vienna, Austria: R Foundation for Statistical Computing; 2017.

[57] Hothorn T, Hornik K, Van De Wiel MA, Zeileis A. Implementing a class of permutation pests: the coin package. Journal of Statistical Software. 2008;28(8):1–23.27774042

[58] Gardner DM, Murphy AL, O’Donnell H, Centorrino F, Baldessarini RJ. International consensus study of antipsychotic dosing. The American journal of psychiatry. 2010;167(6):686–93. 10.1176/appi.ajp.2009.09060802 . 20360319

[59] Filippi M, Cercignani M, Inglese M, Horsfield MA, Comi G. Diffusion tensor magnetic resonance imaging in multiple sclerosis. Neurology. 2001;56(3):304–11. . 11171893 10.1212/wnl.56.3.304

[60] Kubicki M, Shenton ME. Diffusion Tensor Imaging findings and their implications in schizophrenia. Current opinion in psychiatry. 2014;27(3):179–84. 10.1097/YCO.0000000000000053 . 24613986

[61] Aung WY, Mar S, Benzinger TL. Diffusion tensor MRI as a biomarker in axonal and myelin damage. Imaging Med. 2013;5(5):427–40. 10.2217/iim.13.49 . 24795779 PMC4004089

[62] Klawiter EC, Schmidt RE, Trinkaus K, Liang HF, Budde MD, Naismith RT, et al Radial diffusivity predicts demyelination in ex vivo multiple sclerosis spinal cords. Neuroimage. 2011;55(4):1454–60. Epub 2011/01/18. 10.1016/j.neuroimage.2011.01.007 . 21238597 PMC3062747

[63] Wheeler-Kingshott CA, Cercignani M. About "axial" and "radial" diffusivities. Magn Reson Med. 2009;61(5):1255–60. 10.1002/mrm.21965 . 19253405

[64] Cho KI, Shenton ME, Kubicki M, Jung WH, Lee TY, Yun JY, et al Altered Thalamo-Cortical White Matter Connectivity: Probabilistic Tractography Study in Clinical-High Risk for Psychosis and First-Episode Psychosis. Schizophrenia bulletin. 2016;42(3):723–31. 10.1093/schbul/sbv169 . 26598740 PMC4838094

[65] Zhang Y, Su TP, Liu B, Zhou Y, Chou KH, Lo CY, et al Disrupted thalamo-cortical connectivity in schizophrenia: a morphometric correlation analysis. Schizophrenia research. 2014;153(1–3):129–35. 10.1016/j.schres.2014.01.023 . 24529363

[66] Woodward ND, Karbasforoushan H, Heckers S. Thalamocortical dysconnectivity in schizophrenia. The American journal of psychiatry. 2012;169(10):1092–9. 10.1176/appi.ajp.2012.12010056 . 23032387 PMC3810300

[67] Henze R, Brunner R, Thiemann U, Parzer P, Klein J, Resch F, et al The optic radiation and the cerebellar peduncles in adolescents with first-admission schizophrenia—a diffusion tensor imaging study. Journal of neuroimaging: official journal of the American Society of Neuroimaging. 2014;24(2):111–6. 10.1111/j.1552-6569.2011.00668.x . 22268521

[68] Butler PD, Hoptman MJ, Nierenberg J, Foxe JJ, Javitt DC, Lim KO. Visual white matter integrity in schizophrenia. The American journal of psychiatry. 2006;163(11):2011–3. 10.1176/appi.ajp.163.11.2011 . 17074957 PMC1975779

[69] Reavis EA, Lee J, Wynn JK, Narr KL, Njau SN, Engel SA, et al Linking optic radiation volume to visual perception in schizophrenia and bipolar disorder. Schizophr Res. 2017;190:102–6. 10.1016/j.schres.2017.03.027 . 28318839 PMC5600632

[70] Butler PD, Silverstein SM, Dakin SC. Visual perception and its impairment in schizophrenia. Biological psychiatry. 2008;64(1):40–7. 10.1016/j.biopsych.2008.03.023 . 18549875 PMC2435292

[71] Javitt DC, Freedman R. Sensory processing dysfunction in the personal experience and neuronal machinery of schizophrenia. The American journal of psychiatry. 2015;172(1):17–31. 10.1176/appi.ajp.2014.13121691 . 25553496 PMC4501403

[72] Green MF, Lee J, Cohen MS, Engel SA, Korb AS, Nuechterlein KH, et al Functional neuroanatomy of visual masking deficits in schizophrenia. Archives of general psychiatry. 2009;66(12):1295–303. 10.1001/archgenpsychiatry.2009.132 . 19996034 PMC2907419

[73] Makris N, Papadimitriou GM, Kaiser JR, Sorg S, Kennedy DN, Pandya DN. Delineation of the middle longitudinal fascicle in humans: a quantitative, in vivo, DT-MRI study. Cereb Cortex. 2009;19(4):777–85. 10.1093/cercor/bhn124 . 18669591 PMC2651473

[74] Asami T, Saito Y, Whitford TJ, Makris N, Niznikiewicz M, McCarley RW, et al Abnormalities of middle longitudinal fascicle and disorganization in patients with schizophrenia. Schizophrenia research. 2013;143(2–3):253–9. Epub 2013/01/08. 10.1016/j.schres.2012.11.030 . 23290607 PMC3587354

[75] Makris N, Preti MG, Asami T, Pelavin P, Campbell B, Papadimitriou GM, et al Human middle longitudinal fascicle: variations in patterns of anatomical connections. Brain Struct Funct. 2013;218(4):951–68. 10.1007/s00429-012-0441-2 . 22782432 PMC3500586

[76] Wang Y, Fernandez-Miranda JC, Verstynen T, Pathak S, Schneider W, Yeh FC. Rethinking the role of the middle longitudinal fascicle in language and auditory pathways. Cereb Cortex. 2013;23(10):2347–56. 10.1093/cercor/bhs225 . 22875865

[77] Makris N, Zhu A, Papadimitriou GM, Mouradian P, Ng I, Scaccianoce E, et al Mapping temporo-parietal and temporo-occipital cortico-cortical connections of the human middle longitudinal fascicle in subject-specific, probabilistic, and stereotaxic Talairach spaces. Brain Imaging Behav. 2017;11(5):1258–77. Epub 2016/10/08. 10.1007/s11682-016-9589-3 . 27714552 PMC5382125

[78] Kubicki M, Westin CF, Maier SE, Frumin M, Nestor PG, Salisbury DF, et al Uncinate fasciculus findings in schizophrenia: a magnetic resonance diffusion tensor imaging study. The American journal of psychiatry. 2002;159(5):813–20. Epub 2002/05/03. 10.1176/appi.ajp.159.5.813 mc2803760. 11986136 PMC2803760

[79] Kawashima T, Nakamura M, Bouix S, Kubicki M, Salisbury DF, Westin CF, et al Uncinate fasciculus abnormalities in recent onset schizophrenia and affective psychosis: a diffusion tensor imaging study. Schizophrenia research. 2009;110(1–3):119–26. Epub 2009/03/31. 10.1016/j.schres.2009.01.014 . 19328656 PMC2749228

[80] Szoke A, Trandafir A, Dupont ME, Meary A, Schurhoff F, Leboyer M. Longitudinal studies of cognition in schizophrenia: meta-analysis. The British journal of psychiatry: the journal of mental science. 2008;192(4):248–57. 10.1192/bjp.bp.106.029009 . 18378982

[81] Ojeda N, Sanchez P, Pena J, Elizagarate E, Yoller AB, Larumbe J, et al Verbal fluency in schizophrenia: does cognitive performance reflect the same underlying mechanisms in patients and healthy controls? J Nerv Ment Dis. 2010;198(4):286–91. Epub 2010/04/14. 10.1097/NMD.0b013e3181d61748 . 20386258

[82] Silver H, Feldman P, Bilker W, Gur RC. Working memory deficit as a core neuropsychological dysfunction in schizophrenia. The American journal of psychiatry. 2003;160(10):1809–16. 10.1176/appi.ajp.160.10.1809 . 14514495

[83] Lett TA, Voineskos AN, Kennedy JL, Levine B, Daskalakis ZJ. Treating working memory deficits in schizophrenia: a review of the neurobiology. Biological psychiatry. 2014;75(5):361–70. 10.1016/j.biopsych.2013.07.026 . 24011822

[84] Forbes NF, Carrick LA, McIntosh AM, Lawrie SM. Working memory in schizophrenia: a meta-analysis. Psychological medicine. 2009;39(6):889–905. 10.1017/S0033291708004558 . 18945379

[85] Twamley EW, Palmer BW, Jeste DV, Taylor MJ, Heaton RK. Transient and executive function working memory in schizophrenia. Schizophrenia research. 2006;87(1–3):185–90. 10.1016/j.schres.2006.04.013 . 16737800

[86] Viher PV, Stegmayer K, Giezendanner S, Federspiel A, Bohlhalter S, Vanbellingen T, et al Cerebral white matter structure is associated with DSM-5 schizophrenia symptom dimensions. Neuroimage Clin. 2016;12:93–9. Epub 2016/07/14. 10.1016/j.nicl.2016.06.013 . 27408794 PMC4925890

[87] Guo W, Liu F, Liu Z, Gao K, Xiao C, Chen H, et al Right lateralized white matter abnormalities in first-episode, drug-naive paranoid schizophrenia. Neuroscience letters. 2012;531(1):5–9. 10.1016/j.neulet.2012.09.033 . 23022507

[88] Federspiel A, Begre S, Kiefer C, Schroth G, Strik WK, Dierks T. Alterations of white matter connectivity in first episode schizophrenia. Neurobiology of disease. 2006;22(3):702–9. 10.1016/j.nbd.2006.01.015 . 16624566

[89] Nakamura K, Kawasaki Y, Takahashi T, Furuichi A, Noguchi K, Seto H, et al Reduced white matter fractional anisotropy and clinical symptoms in schizophrenia: a voxel-based diffusion tensor imaging study. Psychiatry research. 2012;202(3):233–8. 10.1016/j.pscychresns.2011.09.006 . 22819228

[90] Lee SH, Kubicki M, Asami T, Seidman LJ, Goldstein JM, Mesholam-Gately RI, et al Extensive white matter abnormalities in patients with first-episode schizophrenia: a Diffusion Tensor Iimaging (DTI) study. Schizophrenia research. 2013;143(2–3):231–8. Epub 2013/01/08. 10.1016/j.schres.2012.11.029 . 23290268 PMC4354799

[91] Szeszko PR, Robinson DG, Ikuta T, Peters BD, Gallego JA, Kane J, et al White matter changes associated with antipsychotic treatment in first-episode psychosis. Neuropsychopharmacology: official publication of the American College of Neuropsychopharmacology. 2014;39(6):1324–31. Epub 2014/02/20. 10.1038/npp.2013.288 . 24549105 PMC3988536

[92] Ozcelik-Eroglu E, Ertugrul A, Oguz KK, Has AC, Karahan S, Yazici MK. Effect of clozapine on white matter integrity in patients with schizophrenia: a diffusion tensor imaging study. Psychiatry research. 2014;223(3):226–35. Epub 2014/07/12. 10.1016/j.pscychresns.2014.06.001 . 25012780

[93] Wang Q, Cheung C, Deng W, Li M, Huang C, Ma X, et al White-matter microstructure in previously drug-naive patients with schizophrenia after 6 weeks of treatment. Psychological medicine. 2013;43(11):2301–9. 10.1017/S0033291713000238 . 23442742

[94] Kochunov P, Glahn DC, Rowland LM, Olvera RL, Winkler A, Yang YH, et al Testing the hypothesis of accelerated cerebral white matter aging in schizophrenia and major depression. Biological psychiatry. 2013;73(5):482–91. Epub 2012/12/04. 10.1016/j.biopsych.2012.10.002 23200529 PMC3645491

[95] Mandl RC, Rais M, van Baal GC, van Haren NE, Cahn W, Kahn RS, et al Altered white matter connectivity in never-medicated patients with schizophrenia. Hum Brain Mapp. 2013;34(9):2353–65. Epub 2012/03/31. 10.1002/hbm.22075 . 22461372 PMC6870329

[96] Savadjiev P, Whitford TJ, Hough ME, Clemm von Hohenberg C, Bouix S, Westin CF, et al Sexually dimorphic white matter geometry abnormalities in adolescent onset schizophrenia. Cereb Cortex. 2014;24(5):1389–96. 10.1093/cercor/bhs422 . 23307635 PMC3977625

[97] Vos SB, Aksoy M, Han Z, Holdsworth SJ, Maclaren J, Viergever MA, et al Trade-off between angular and spatial resolutions in in vivo fiber tractography. Neuroimage. 2016;129:117–32. 10.1016/j.neuroimage.2016.01.011 . 26774615 PMC4803623

[98] Yendiki A, Koldewyn K, Kakunoori S, Kanwisher N, Fischl B. Spurious group differences due to head motion in a diffusion MRI study. Neuroimage. 2014;88:79–90. 10.1016/j.neuroimage.2013.11.027 . 24269273 PMC4029882

