# Supplementary material for: Altered white matter connectivity in patients with schizophrenia: An investigation using public neuroimaging data from SchizConnect
Source: PLoS One. 2018 Oct 9;13(10):e0205369. doi: 10.1371/journal.pone.0205369 (PMC6177186; doi:10.1371/journal.pone.0205369)
Supplement: S1 Table — COBRE (Center of Biomedical Research Excellence), MCIC (Mind Clinical Imaging Consortium), NmorphCH (Neuromorphometry by Computer Algorithm Chicago), DWI (diffusion weighted imaging). (DOCX) [file pone.0205369.s001.docx]

**S1 Table. Scanners and imaging parameters by sample**

| **Study** | **Sequence** | **Scanners** | **Protocol parameters** |  |
| --- | --- | --- | --- | --- |
| COBRE | T1 | 3T Siemens Trio | Coronal T1-weighted structural images were acquired with a 12-channel head-coil and a five-echo MPRAGE sequence (TE = 1.64, 3.5, 5.36, 7.22, and 9.08 ms; TR = 2.53 s; TI = 1.2 s, flip angle = 7°; number of excitations = 1; slice thickness = 1 mm; field of view = 256 mm; resolution = 256 × 256) |  |
| COBRE | DWI | 3T Siemens Trio | DWI images were acquired with a voxel size of 2.0 × 2.0 × 2.0 mm (TR = 9000 ms; TE = 84 ms; B values of 0 and 800; bandwidth = 1562; 72 slices and 35 directions) |  |
| MCIC | T1 | 3T Siemens SMS Trio | Coronal T1 scans were collected with an eight-channel head coil and a gradient echo sequence (TR = 12 ms; TE = 3.79 ms; flip angle = 20°; bandwidth = 181; voxel size = 0.625 × 0.625 mm; slice thickness = 1.5 mm; matrix = 256 × 256; number of slices = 128) |  |
| MCIC | | DWI | 3T Siemens SMS Trio | DWI images were acquired with a slice thickness of 2 mm, with a 2 mm isotropic resolution (TR = 10500 ms; TE = 98 ms; B values of 0 and 1000; NEX = 2; bandwidth = 1342; 64 slices and 12 directions) |
| NMorphCH | | T1 | 3T Siemens Trio | Coronal T1-weighted structural images were acquired with a 32-channel head coil and a MPRAGE sequence (TR = 2400 ms, TE = 3.16 ms, flip = 8°, 256 × 256 matrix, 176 slices, slice thickness = 1 mm) |
| NMorphCH | | DWI | 3T Siemens Trio | DWI images were acquired with a slice thickness of 2 mm (TR = 8000 ms, TE = 86 ms, B values of 0 and 800; flip = 90°, 896 × 896 matrix, 35 slices and 30 directions) |

Note: COBRE (Center of Biomedical Research Excellence), MCIC (Mind Clinical Imaging Consortium), NMorphCH (Neuromorphometry by Computer Algorithm Chicago), DWI (diffusion weighted imaging).
