# Supplementary material for: Altered white matter connectivity in patients with schizophrenia: An investigation using public neuroimaging data from SchizConnect
Source: PLoS One. 2018 Oct 9;13(10):e0205369. doi: 10.1371/journal.pone.0205369 (PMC6177186; doi:10.1371/journal.pone.0205369)
Supplement: S2 Table — AF (arcuate fascicle), CB (cingulum bundle), CC (corpus callosum), CST (corticospinal tract), EC (external capsule), ILF (inferior longitudinal fascicle), IC (internal capsule), IOFF (inferior occipitofrontal fascicle), MDLF (middle longitudinal fascicle), SLF (superior longitudinal fascicle), SOFF (superior occipitofrontal fascicle), UF (uncinate fascicle). (DOCX) [file pone.0205369.s002.docx]

**S2 Table. Anatomical definitions of white matter query language** [1]

| **Structure** | **White matter query language query** |
| --- | --- |
| AF | AF.side = (inferior_frontal_gyrus.side or middle_frontal_gyrus.side or precentral.side) and (superiortemporal.side or middletemporal.side) not in hemisphere.opposite, medial_of(supramarginal.side), ilf.side, ioff.side, ec.side, mdlf.side, mdlf_probable_sections.side, temporalpole.side, frontalpole.side, subcortical.side, rostralmiddlefrontal.side, lateralorbitofrontal.side, parstriangularis.side, superiorfrontal.side, parsopercularis.side |
| CB | CB.side = only(cingular.side or cingular_cortex.side and (middle_frontal.side or cuneus.side or entorhinal.side or superior_frontal.side or inferior parietal.side or fusiform.side or medial orbitofrontal.side or lateral orbitofrontal.side or unsegmentedwhitematter.side or parahippocampal.side or precuneus.side or lingual.side)) |
| CC | cc = cc_1 or cc_2 or cc_3 or cc_4 or cc_5 or cc_6 or cc_7  cc_1 = endpoints_in(orbitofrontalgyrus.left) and endpoints_in(orbitofrontalgyrus.right)  cc_2 = endpoints_in(parstriangularis.left or parsorbitalis.left or superiorfrontal.left or rostralmiddlefrontal.left or frontalpole.left) and endpoints_in (parstriangularis.right or parsorbitalis.right or superiorfrontal.right or rostralmiddlefrontal.right or frontalpole.right)  cc_3 = endpoints_in(rostralanteriorcingulate.left or caudalanteriorcingulate.left or caudalmiddlefrontal.left or parsopercularis.left) and endpoints_in(rostralanteriorcingulate.right or caudalanteriorcingulate.right or caudalmiddlefrontal.right or parsopercularis.right)  cc_4 = endpoints_in(precentral.left) and endpoints_in(precentral.right)  cc_5 = endpoints_in(postcentral.left or posteriorcingulate.left or paracentral.left) and endpoints_in(postcentral.right or posteriorcingulate.right or paracentral.right)  cc_6 = endpoints_in(precuneus.left or supramarginal.left or superiorparietal.left or inferiorparietal.left or superiortemporal.left or middletemporal.left or bankssts.left or transversetemporal.left or isthmuscingulate.left) and endpoints_in(precuneus.right or supramarginal.right or superiorparietal.right or inferiorparietal.right or superiortemporal.right or middletemporal.right or bankssts.right or transversetemporal.right or isthmuscingulate.right)  cc_7 = endpoints_in(occipital.left or fusiform.left or lingual.left or inferiortemporal.left) and endpoints_in(occipital.right or fusiform.right or lingual.right or inferiortemporal.right) |
| CST | cortico_spinal.side = endpoints_in(brainstem) and endpoints_in(precentral.side or postcentral.side) not in superiorfrontal.side, cerebellum_white_matter.side, thalamus_proper.side, hemisphere.opposite |
| EC | EC.side = (superiortemporal.side and frontal.side) and (parietal.side or occipital.side) not in ioff.side, ilf.side, hemisphere.opposite, subcortical.side, uf.side, inferiortemporal.side, fusiform.side, middletemporal.side, temporalpole.side, corpuscallosum.side, precentral.side, parsopercularis.side, cingular.side |
| ILF | ILF.side = only(temporal.side and occipital.side) and anterior_of(hippocampus.side) not in parahippocampal.side |
| IC | IC.side = ((frontal.side and thalamus_proper.side and (parietal.side or occipital.side)) not in cingular.side, precentral.side, postcentral.side, hemisphere.opposite, brain_stem ) |
| IOFF | IOFF.side = endpoints_in(orbitofrontalgyrus.side or inferiorfrontalgyrus.side) and endpoints_in(occipital.side) and temporal.side and insula.side |
| MDLF | MDLF.side = only((temporalpole.side or superiortemporal.side) and (inferiorparietal.side or superiorparietal.side or supramarginal.side or precuneus.side or (unsegmentedwhitematter.side and superiorparietal.side) or (unsegmentedwhitematter.side and inferiorparietal.side))) |
| SLF i | SLF_I.side = (superiorparietal.side and precuneus.side and superiorfrontal.side) or (superiorparietal.side and precuneus.side and superiorfrontal.side and lateraloccipital.side) not in cingular.side, temporal.side, subcortical.side, hemisphere.opposite |
| SLF ii | SLF_II.side = (lateraloccipital.side or supramarginal.side or inferiorparietal.side) and endpoints_in(middle_frontal_gyrus.side)) not in hemisphere.opposite, temporal.side, cingular.side, subcortical.side |
| SLF iii | SLF_III.side = (lateraloccipital.side or supramarginal.side or inferiorparietal.side) and endpoints_in(inferior_frontal_gyrus.side) not in hemisphere.opposite, temporal.side, cingular.side, subcortical.side |
| SOFF | SOFF.side = (lateralorbitofrontal.side and occipital.side) not in temporal.side, subcortical.side, cingular.side, hemisphere.opposite |
| striato-frontal | striato_frontal.side = endpoints_in(striatum.side) and endpoints_in(frontal.side) |
| striato-occipital | striato_occipital.side = endpoints_in(striatum.side) and endpoints_in(occipital.side) |
| striato-parietal | striato_parietal.side = endpoints_in(striatum.side) and endpoints_in(parietal.side) |
| thalamo-frontal | thalamo_frontal.side = endpoints_in(thalamus.side) and endpoints_in(frontal.side) |
| thalamo-occipital | thalamo_occipital.side = endpoints_in(thalamus.side) and endpoints_in(occipital.side) |
| thalamo-parietal | thalamo_parietal.side = endpoints_in(thalamus.side) and endpoints_in(parietal.side) |
| UF | UF.side = (orbitofrontalgyrus.side or inferior_frontal_gyrus.side) and endpoints_in(temporalpole.side and insula.side) not in occipital.side, parietal.side, cingular.side, posterior_of(putamen.side), hemisphere.opposite |

Note: AF (arcuate fascicle), CB (cingulum bundle), CC (corpus callosum), CST (corticospinal tract), EC (external capsule), ILF (inferior longitudinal fascicle), IC (internal capsule), IOFF (inferior occipitofrontal fascicle), MDLF (middle longitudinal fascicle), SLF (superior longitudinal fascicle), SOFF (superior occipitofrontal fascicle), UF (uncinate fascicle).

**Reference**

1. Wassermann D, Makris N, Rathi Y, Shenton M, Kikinis R, Kubicki M, et al. The white matter query language: a novel approach for describing human white matter anatomy. Brain Struct Funct. 2016;221(9):4705-21. doi: 10.1007/s00429-015-1179-4. PubMed PMID: 26754839; PubMed Central PMCID: PMCPMC4940319.
