# Supplementary material for: Altered white matter connectivity in patients with schizophrenia: An investigation using public neuroimaging data from SchizConnect
Source: PLoS One. 2018 Oct 9;13(10):e0205369. doi: 10.1371/journal.pone.0205369 (PMC6177186; doi:10.1371/journal.pone.0205369)
Supplement: S3 Table — AF (arcuate fascicle), CB (cingulum bundle), CST (corticospinal tract), EC (external capsule), ILF (inferior longitudinal fascicle), IC (internal capsule), IOFF (inferior occipitofrontal fascicle), MDLF (middle longitudinal fascicle), SLF (superior longitudinal fascicle), SOFF (superior occipitofrontal fascicle), UF (uncinate fascicle), AD (axial diffusivity), FA (fractional anisotropy), RD (radial diffusivity), TR (trace). (DOCX) [file pone.0205369.s003.docx]

**S3.1 Table. Group differences in the diffusion measures of tracts in the Center of Biomedical Research Excellence sample^a^**

| **Structure** | **Diffusion measure** | | | | | | | |
| --- | --- | --- | --- | --- | --- | --- | --- | --- |
|  | **AD** | | **FA** | | **RD** | | **TR** | |
|  | ***z*** | ***p***^b^ | ***z*** | ***p***^b^ | ***z*** | ***p***^b^ | ***z*** | ***p***^b^ |
| Corpus callosum | −0.533 | 0.597 | 0.880 | 0.385 | −1.246 | 0.213 | −1.231 | 0.220 |

| **Structure** | **Diffusion measure** | | | | | | | |
| --- | --- | --- | --- | --- | --- | --- | --- | --- |
|  | **AD** | | **FA** | | **RD** | | **TR** | |
|  | ***z*** | ***p***^b^ | ***z*** | ***p***^b^ | ***z*** | ***p***^b^ | ***z*** | ***p***^b^ |
| left AF | −0.073 | 0.941 | 1.176 | 0.243 | −1.164 | 0.247 | −0.782 | 0.434 |
| left CB | 0.979 | 0.329 | 1.576 | 0.114 | −1.104 | 0.270 | −0.420 | 0.676 |
| left CST | 0.261 | 0.796 | 0.886 | 0.378 | −0.368 | 0.715 | 0.451 | 0.655 |
| left EC | 1.206 | 0.227 | 1.906 | 0.057 | −1.913 | 0.056 | −1.382 | 0.167 |
| left ILF | −0.432 | 0.668 | 1.176 | 0.244 | −1.799 | 0.072 | −1.775 | 0.075 |
| left IC | 0.242 | 0.808 | 2.200 | 0.027 | −2.067 | 0.039 | −1.227 | 0.221 |
| left IOFF | 1.334 | 0.184 | 0.406 | 0.687 | −0.692 | 0.491 | 0.970 | 0.341 |
| left MDLF | 1.338 | 0.185 | 2.492 | 0.012 | −2.221 | 0.026 | −1.119 | 0.268 |
| left SLF i | 1.854 | 0.064 | 2.590 | 0.009 | −1.931 | 0.053 | −0.943 | 0.348 |
| left SLF ii | 1.350 | 0.178 | 1.770 | 0.078 | −0.786 | 0.435 | 0.128 | 0.901 |
| left SLF iii | 1.972 | 0.048 | 1.468 | 0.145 | −0.583 | 0.561 | 0.183 | 0.856 |
| left SOFF | 1.366 | 0.176 | −1.309 | 0.195 | 1.067 | 0.290 | 1.703 | 0.089 |
| left striato-frontal | 0.825 | 0.408 | 0.179 | 0.858 | −0.900 | 0.371 | 0.006 | 0.995 |
| left striato-occipital | −0.854 | 0.397 | −0.186 | 0.854 | −0.931 | 0.364 | −0.974 | 0.338 |
| left striato-parietal | 1.148 | 0.254 | 0.889 | 0.374 | −0.192 | 0.851 | 0.507 | 0.615 |
| left thalamo-frontal | 0.752 | 0.456 | 0.354 | 0.725 | 0.220 | 0.829 | 0.195 | 0.847 |
| left thalamo-occipital | −1.981 | 0.047 | 0.064 | 0.950 | −1.593 | 0.112 | −2.057 | 0.038 |
| left thalamo-parietal | 0.602 | 0.551 | 1.025 | 0.309 | −0.622 | 0.540 | −0.197 | 0.848 |
| left UF | 0.032 | 0.975 | 0.014 | 0.989 | 0.781 | 0.437 | 0.643 | 0.525 |

| **Structure** | **Diffusion measure** | | | | | | | |
| --- | --- | --- | --- | --- | --- | --- | --- | --- |
|  | **AD** | | **FA** | | **RD** | | **TR** | |
|  | ***z*** | ***p***^b^ | ***z*** | ***p***^b^ | ***z*** | ***p***^b^ | ***z*** | ***p***^b^ |
| right AF | 1.195 | 0.235 | 1.405 | 0.162 | −1.071 | 0.287 | −0.061 | 0.953 |
| right CB | 2.467 | 0.013 | 1.690 | 0.091 | −0.447 | 0.657 | 0.616 | 0.540 |
| right CST | 0.028 | 0.978 | 1.404 | 0.159 | −0.999 | 0.321 | 0.085 | 0.932 |
| right EC | −0.034 | 0.973 | 0.642 | 0.523 | −0.351 | 0.728 | −0.182 | 0.855 |
| right ILF | 0.495 | 0.623 | 1.712 | 0.087 | −1.563 | 0.119 | −1.052 | 0.302 |
| right IC | 1.033 | 0.302 | 0.869 | 0.385 | −0.08 | 0.935 | 0.477 | 0.637 |
| right IOFF | −0.898 | 0.371 | 2.391 | 0.016 | −2.245 | 0.024 | −0.609 | 0.546 |
| right MDLF | 1.197 | 0.233 | 2.581 | 0.010 | −2.964 | 0.003 | −2.041 | 0.041 |
| right SLF i | 2.176 | 0.030 | 0.807 | 0.423 | 0.403 | 0.688 | 1.192 | 0.237 |
| right SLF ii | 1.125 | 0.260 | 2.311 | 0.021 | −1.796 | 0.073 | −0.911 | 0.363 |
| right SLF iii | 0.919 | 0.361 | 1.665 | 0.095 | −1.670 | 0.097 | −0.956 | 0.342 |
| right SOFF | 0.681 | 0.501 | 1.976 | 0.048 | −1.470 | 0.142 | −1.350 | 0.179 |
| right striato-frontal | 1.596 | 0.112 | 1.079 | 0.281 | −1.134 | 0.257 | 0.444 | 0.659 |
| right striato-occipital | 0.723 | 0.473 | −0.038 | 0.969 | 0.713 | 0.478 | 0.790 | 0.435 |
| right striato-parietal | 1.102 | 0.272 | 1.601 | 0.110 | −0.800 | 0.426 | −0.677 | 0.500 |
| right thalamo-frontal | 0.883 | 0.379 | 2.572 | 0.009 | −1.544 | 0.124 | −0.608 | 0.549 |
| right thalamo-occipital | 1.518 | 0.128 | 0.608 | 0.546 | 0.575 | 0.569 | 1.177 | 0.246 |
| right thalamo-parietal | 0.544 | 0.589 | 0.233 | 0.818 | −0.340 | 0.737 | 0.543 | 0.591 |
| right UF | 0.157 | 0.875 | 2.164 | 0.030 | −2.949 | 0.003 | −2.649 | 0.007 |

Note: AF (arcuate fascicle), CB (cingulum bundle), CST (corticospinal tract), EC (external capsule), ILF (inferior longitudinal fascicle), IC (internal capsule), IOFF (inferior occipitofrontal fascicle), MDLF (middle longitudinal fascicle), SLF (superior longitudinal fascicle), SOFF (superior occipitofrontal fascicle), UF (uncinate fascicle), AD (axial diffusivity), FA (fractional anisotropy), RD (radial diffusivity), TR (trace).

^a^ Approximate Monte Carlo Fisher–Pitman Tests were performed to reveal group differences in the z-scores of the diffusion measures of tracts in the right hemisphere.

^b^ Uncorrected p-values.

**S3.2 Table. Group differences in the diffusion measures of tracts in the Mind Clinical Imaging Consortium sample^a^**

| **Structure** | **Diffusion measure** | | | | | | | |
| --- | --- | --- | --- | --- | --- | --- | --- | --- |
|  | **AD** | | **FA** | | **RD** | | **TR** | |
|  | ***z*** | ***p***^b^ | ***z*** | ***p***^b^ | ***z*** | ***p***^b^ | ***z*** | ***p***^b^ |
| Corpus callosum | −0.431 | 0.669 | −0.206 | 0.829 | −1.135 | 0.264 | −0.450 | 0.660 |

| **Structure** | **Diffusion measure** | | | | | | | |
| --- | --- | --- | --- | --- | --- | --- | --- | --- |
|  | **AD** | | **FA** | | **RD** | | **TR** | |
|  | ***z*** | ***p***^b^ | ***z*** | ***p***^b^ | ***z*** | ***p***^b^ | ***z*** | ***p***^b^ |
| left AF | −0.704 | 0.631 | −0.732 | 0.473 | −1.179 | 0.245 | −0.827 | 0.476 |
| left CB | −0.539 | 0.613 | 1.176 | 0.253 | −1.097 | 0.290 | −0.887 | 0.390 |
| left CST | −0.310 | 0.783 | 0.175 | 0.868 | −0.545 | 0.600 | 0.314 | 0.764 |
| left EC | −0.998 | 0.331 | −0.291 | 0.787 | −1.078 | 0.286 | −1.058 | 0.313 |
| left ILF | 0.426 | 0.680 | 1.998 | 0.045 | −2.206 | 0.024 | −1.409 | 0.164 |
| left IC | −0.670 | 0.511 | −0.593 | 0.565 | −0.743 | 0.466 | −0.697 | 0.498 |
| left IOFF | −0.586 | 0.636 | 0.314 | 0.763 | −0.191 | 0.848 | −1.318 | 0.203 |
| left SLF ii | −1.099 | 0.330 | 1.332 | 0.195 | −2.275 | 0.014 | −2.471 | 0.007 |
| left SLF iii | 1.186 | 0.241 | −0.743 | 0.491 | 0.388 | 0.734 | −1.350 | 0.181 |
| left SOFF | −0.614 | 0.607 | 1.997 | 0.042 | −1.645 | 0.102 | −1.273 | 0.193 |
| left striato-frontal | −0.926 | 0.373 | 0.352 | 0.788 | −1.228 | 0.229 | −1.649 | 0.100 |
| left striato-occipital | 0.788 | 0.484 | 0.972 | 0.341 | 0.220 | 0.923 | 0.484 | 0.779 |
| left striato-parietal | 1.407 | 0.164 | 2.360 | 0.014 | −2.409 | 0.013 | −0.919 | 0.363 |
| left thalamo-frontal | −1.192 | 0.242 | −1.249 | 0.284 | −1.418 | 0.162 | −0.976 | 0.339 |
| left thalamo-occipital | −0.500 | 0.622 | 1.934 | 0.049 | −1.700 | 0.089 | −1.420 | 0.157 |
| left thalamo-parietal | −0.831 | 0.415 | 0.911 | 0.372 | −1.526 | 0.129 | −1.536 | 0.127 |
| left UF | −1.587 | 0.115 | 0.277 | 0.731 | −1.965 | 0.048 | −2.053 | 0.038 |

| **Structure** | **Diffusion measure** | | | | | | | |
| --- | --- | --- | --- | --- | --- | --- | --- | --- |
|  | **AD** | | **FA** | | **RD** | | **TR** | |
|  | ***z*** | ***p***^b^ | ***z*** | ***p***^b^ | ***z*** | ***p***^b^ | ***z*** | ***p***^b^ |
| right AF | −1.037 | 0.313 | −0.299 | 0.768 | −1.461 | 0.145 | −1.424 | 0.160 |
| right CB | −0.384 | 0.707 | −0.146 | 0.956 | −2.144 | 0.021 | −0.845 | 0.404 |
| right CST | −1.058 | 0.297 | −0.343 | 0.737 | −0.918 | 0.366 | −1.134 | 0.267 |
| right EC | −1.016 | 0.370 | −0.637 | 0.524 | −1.998 | 0.045 | −1.111 | 0.269 |
| right ILF | 0.806 | 0.426 | 2.350 | 0.017 | −2.416 | 0.014 | −1.585 | 0.117 |
| right IC | −0.956 | 0.385 | −0.329 | 0.755 | −0.967 | 0.355 | −1.052 | 0.318 |
| right IOFF | −0.534 | 0.599 | −1.474 | 0.147 | −0.054 | 0.957 | −0.779 | 0.446 |
| right MDLF | −0.033 | 0.975 | 0.951 | 0.350 | −1.122 | 0.271 | −1.033 | 0.312 |
| right SLF i | −1.454 | 0.150 | −0.654 | 0.529 | −1.778 | 0.066 | −1.760 | 0.080 |
| right SLF ii | 0.086 | 0.935 | 0.105 | 0.918 | −2.128 | 0.025 | −1.412 | 0.161 |
| right SLF iii | 0.424 | 0.718 | 1.967 | 0.047 | −0.582 | 0.588 | −0.176 | 0.918 |
| right SOFF | 0.085 | 0.933 | 0.034 | 0.975 | −0.250 | 0.806 | −1.007 | 0.318 |
| right striato-frontal | −0.624 | 0.507 | −0.342 | 0.998 | −0.114 | 0.915 | −1.019 | 0.311 |
| right striato-occipital | −0.910 | 0.383 | −0.880 | 0.394 | −0.055 | 0.958 | −0.503 | 0.625 |
| right striato-parietal | −0.999 | 0.493 | 0.797 | 0.433 | −0.473 | 0.642 | −1.112 | 0.212 |
| right thalamo-frontal | −0.521 | 0.611 | −0.204 | 0.963 | −2.144 | 0.025 | −1.307 | 0.124 |
| right thalamo-occipital | −0.188 | 0.856 | 1.300 | 0.203 | −1.471 | 0.149 | −1.178 | 0.244 |
| right thalamo-parietal | −1.881 | 0.057 | 0.668 | 0.514 | −0.785 | 0.448 | −1.854 | 0.062 |
| right UF | −0.132 | 0.895 | 0.969 | 0.339 | −1.577 | 0.116 | −0.929 | 0.362 |

Note: AF (arcuate fascicle), CB (cingulum bundle), CST (corticospinal tract), EC (external capsule), ILF (inferior longitudinal fascicle), IC (internal capsule), IOFF (inferior occipitofrontal fascicle), MDLF (middle longitudinal fascicle), SLF (superior longitudinal fascicle), SOFF (superior occipitofrontal fascicle), UF (uncinate fascicle), AD (axial diffusivity), FA (fractional anisotropy), RD (radial diffusivity), TR (trace).

^a^ Approximate Monte Carlo Fisher–Pitman Tests were performed to reveal group differences in the z-scores of the diffusion measures of tracts in the right hemisphere.

^b^ Uncorrected p-values.

**S3.3 Table. Group differences in the diffusion measures of tracts in the Neuromorphometry by Computer Algorithm Chicago sample^a^**

| **Structure** | **Diffusion measure** | | | | | | | |
| --- | --- | --- | --- | --- | --- | --- | --- | --- |
|  | **AD** | | **FA** | | **RD** | | **TR** | |
|  | ***z*** | ***p***^b^ | ***z*** | ***p***^b^ | ***z*** | ***p***^b^ | ***z*** | ***p***^b^ |
| Corpus callosum | −0.715 | 0.479 | −1.194 | 0.232 | 0.788 | 0.434 | 0.298 | 0.768 |

| **Structure** | **Diffusion measure** | | | | | | | |
| --- | --- | --- | --- | --- | --- | --- | --- | --- |
|  | **AD** | | **FA** | | **RD** | | **TR** | |
|  | ***z*** | ***p***^b^ | ***z*** | ***p***^b^ | ***z*** | ***p***^b^ | ***z*** | ***p***^b^ |
| left AF | 0.735 | 0.466 | 0.218 | 0.827 | −0.400 | 0.694 | 0.469 | 0.644 |
| left CB | −0.011 | 0.992 | 0.342 | 0.735 | −0.403 | 0.692 | −0.325 | 0.746 |
| left CST | 0.020 | 0.985 | −0.450 | 0.664 | 0.177 | 0.863 | 0.382 | 0.704 |
| left EC | 0.437 | 0.669 | 1.572 | 0.117 | −1.592 | 0.112 | −0.272 | 0.786 |
| left ILF | 1.159 | 0.250 | 1.530 | 0.129 | −1.201 | 0.231 | −0.267 | 0.792 |
| left IC | 0.585 | 0.566 | 0.911 | 0.366 | −0.460 | 0.651 | −0.011 | 0.991 |
| left IOFF | −0.079 | 0.940 | 0.174 | 0.864 | −0.377 | 0.710 | 0.060 | 0.955 |
| left MDLF | 0.488 | 0.626 | 0.809 | 0.421 | −0.994 | 0.326 | −0.319 | 0.747 |
| left SLF i | 0.148 | 0.884 | 0.513 | 0.610 | −0.389 | 0.708 | −0.218 | 0.836 |
| left SLF ii | 0.418 | 0.684 | 0.524 | 0.603 | −0.426 | 0.678 | −0.117 | 0.915 |
| left SLF iii | 0.194 | 0.848 | −1.275 | 0.205 | 1.559 | 0.121 | 1.031 | 0.306 |
| left SOFF | −0.107 | 0.918 | −1.645 | 0.100 | 2.437 | 0.014 | 0.475 | 0.649 |
| left striato-frontal | 0.307 | 0.762 | 1.542 | 0.124 | −1.047 | 0.297 | −0.246 | 0.804 |
| left striato-occipital | −0.990 | 0.332 | −0.035 | 0.971 | −1.433 | 0.153 | −1.326 | 0.188 |
| left striato-parietal | 0.558 | 0.581 | −0.923 | 0.362 | 1.251 | 0.212 | 1.152 | 0.253 |
| left thalamo-frontal | 1.171 | 0.241 | 0.720 | 0.477 | 0.070 | 0.945 | 0.506 | 0.616 |
| left thalamo-occipital | −0.258 | 0.806 | 2.540 | 0.009 | −2.773 | 0.004 | −1.850 | 0.063 |
| left thalamo-parietal | <0.001 | 1.000 | −1.101 | 0.274 | 0.836 | 0.408 | 0.567 | 0.574 |
| left UF | −2.647 | 0.007 | −0.214 | 0.831 | −2.057 | 0.039 | −2.991 | 0.002 |

| **Structure** | **Diffusion measure** | | | | | | | |
| --- | --- | --- | --- | --- | --- | --- | --- | --- |
|  | **AD** | | **FA** | | **RD** | | **TR** | |
|  | ***z*** | ***p***^b^ | ***z*** | ***p***^b^ | ***z*** | ***p***^b^ | ***z*** | ***p***^b^ |
| right AF | −1.258 | 0.211 | −1.005 | 0.319 | 0.323 | 0.749 | −0.753 | 0.456 |
| right CB | 0.438 | 0.662 | −0.702 | 0.487 | 0.910 | 0.369 | 0.497 | 0.621 |
| right CST | 0.223 | 0.826 | −0.035 | 0.973 | −0.168 | 0.869 | −0.330 | 0.742 |
| right EC | 0.352 | 0.729 | 0.969 | 0.336 | −0.992 | 0.323 | −0.970 | 0.336 |
| right ILF | −0.266 | 0.793 | 0.114 | 0.911 | −0.424 | 0.674 | −0.524 | 0.606 |
| right IC | 1.285 | 0.199 | 1.676 | 0.095 | −0.637 | 0.524 | 0.152 | 0.880 |
| right IOFF | −0.278 | 0.783 | 1.321 | 0.193 | −2.227 | 0.025 | −2.185 | 0.028 |
| right MDLF | 0.412 | 0.682 | 0.799 | 0.427 | −0.918 | 0.362 | −0.589 | 0.558 |
| right SLF i | 0.804 | 0.427 | 1.534 | 0.124 | −1.256 | 0.211 | −0.674 | 0.504 |
| right SLF ii | 0.782 | 0.435 | 1.515 | 0.131 | −1.230 | 0.220 | −0.682 | 0.498 |
| right SLF iii | 0.689 | 0.494 | 0.979 | 0.330 | −0.658 | 0.513 | −0.167 | 0.868 |
| right SOFF | −0.128 | 0.900 | 0.274 | 0.789 | −0.579 | 0.567 | 0.602 | 0.552 |
| right striato-frontal | 1.162 | 0.248 | 0.910 | 0.367 | −0.593 | 0.555 | 0.403 | 0.689 |
| right striato-occipital | 0.738 | 0.478 | 0.019 | 0.985 | −0.129 | 0.897 | 0.311 | 0.763 |
| right striato-parietal | 0.826 | 0.410 | 1.555 | 0.120 | −1.345 | 0.180 | −0.771 | 0.444 |
| right thalamo-frontal | 0.825 | 0.413 | −0.465 | 0.646 | 0.906 | 0.369 | 0.979 | 0.330 |
| right thalamo-occipital | −0.468 | 0.670 | 1.653 | 0.099 | −2.312 | 0.013 | −1.597 | 0.101 |
| right thalamo-parietal | −0.513 | 0.608 | 0.348 | 0.730 | −1.106 | 0.271 | −0.687 | 0.495 |
| right UF | −0.416 | 0.681 | 1.347 | 0.178 | −2.246 | 0.023 | −2.248 | 0.024 |

Note: AF (arcuate fascicle), CB (cingulum bundle), CST (corticospinal tract), EC (external capsule), ILF (inferior longitudinal fascicle), IC (internal capsule), IOFF (inferior occipitofrontal fascicle), MDLF (middle longitudinal fascicle), SLF (superior longitudinal fascicle), SOFF (superior occipitofrontal fascicle), UF (uncinate fascicle), AD (axial diffusivity), FA (fractional anisotropy), RD (radial diffusivity), TR (trace).

^a^ Approximate Monte Carlo Fisher–Pitman Tests were performed to reveal group differences in the z-scores of the diffusion measures of tracts in the right hemisphere.

^b^ Uncorrected p-values.
